# The potential of cellular homing behavior in tumor immunotherapy: from basic discoveries to clinical applications of immune, mesenchymal stem, and cancer cell homing

**DOI:** 10.3389/fimmu.2024.1495978

**Published:** 2024-12-12

**Authors:** Dongtao Li, Yixuan Yang, Guangda Zheng, Linghan Meng, Lu Shang, Juanxia Ren, Lingyun Wang, Yanju Bao

**Affiliations:** ^1^ Department of Oncology, Guang’anmen Hospital, China Academy of Chinese Medical Sciences, Beijing, China; ^2^ First Clinical College, Liaoning University of Traditional Chinese Medicine, Shenyang, Liaoning, China

**Keywords:** immunotherapy, tumor, tumor microenvironment, cell homing, treatment strategies

## Abstract

The efficacy of immunotherapy, a pivotal approach in the arsenal of cancer treatment strategies, is contingent on the capacity of effector cells to localize at the tumor site. The navigational capacity of these cells is intricately linked to the homing behaviors of specific cell types. Recent studies have focused on leveraging immune cells and mesenchymal stem cells (MSCs) homing for targeted tumor therapy and incorporating cancer cell homing properties into anti-tumor strategies. However, research and development of immunotherapy based on cancer cell homing remain in their preliminary stages. Enhancing the homing efficiency of effector cells is essential; therefore, understanding the underlying mechanisms and addressing immune resistance within the tumor microenvironment and challenges associated with *in vivo* therapeutic agent delivery are essential. This review firstly delineates the discovery and clinical translation of the three principal cell-homing behaviors. Secondly, we endeavor to conduct an in-depth analysis of existing research on the homing of immune and stem cells in cancer therapy, with the aim of identifying and understanding of the common applications, potential benefits, barriers, and critical success factors of cellular homing therapies. Finally, based on the understanding of the key factors of cellular homing therapies, we provide an overview and outlook on the enormous potential of harnessing cancer cells’ self-homing to treat tumors. Although immunotherapy based on cell-homing behavior warrants further research, it remains a highly competitive treatment modality that can be combined with existing classic anti-cancer therapies. In general, combining the homing properties of cells to optimize their clinical effects is also one of the future research directions in the field of cell transplantation.

## 1 Introduction

Cell homing, a critical cellular migration process, significantly affects organismal development, tissue regeneration, and disease progression. The term “homing” was initially coined to describe the tendency of lymphocytes circulating within the bloodstream to migrate toward their site of origin, such as lymph nodes, akin to birds returning to their nests. This concept was introduced by Gallatin in 1983 (1). Subsequently, the concept of “homing” was extended to include stem cell migration (2). Stem cell homing refers to the directed migration of endogenous or exogenous stem cells under the influence of various factors, enabling them to traverse vascular endothelial cells and colonize target tissues (3). This process is analogous to the migration of white blood cells to the sites of inflammation in the human body. In 2009, Krap et al. defined “mesenchymal stem cell homing” as the process by which mesenchymal stem cells (MSCs) are captured within the vascular network of the target tissue and subsequently migrate across vascular endothelial cells to the target tissue (4). In 2010, Saito et al. proposed that MSCs possessed homing capabilities. Upon stimulation by specific triggers, previously “quiescent” MSCs are “activated” and migrate back to the injury site to differentiate and replace damaged tissue (5). The homing ability of stem cells can be likened to an intrinsic GPS, autonomously navigating to sites of cellular repair within the body and facilitating timely tissue regeneration.

Malignant tumors are diseases in which normal cells undergo genetic mutations induced by genetic or environmental factors, culminating in uncontrolled cellular growth, migration, invasion, dysregulation of apoptosis, and metabolic anomalies. These neoplasms pose a significant threat to public health and cause a multitude of adverse biological manifestations (6). The conventional clinical management of cancer encompasses radiotherapy, chemotherapy, surgical resection, and novel biological therapies aimed at targeting tumor-specific molecules or stimulating the immune system. Despite these interventions, the five-year survival rates of patients remain poor (7). Immunotherapy, a burgeoning field in oncology, has attracted significant attention. Yet, its clinical utility remains limited, primarily due to the formidable nature of the tumor microenvironment (TME), which suppresses immune responses, making entry into tumor tissues difficult for killers, such as CAR-T cells (8). Consequently, it is imperative to delineate novel and efficacious therapeutic strategies for cancer treatment.

**Figure 1:**
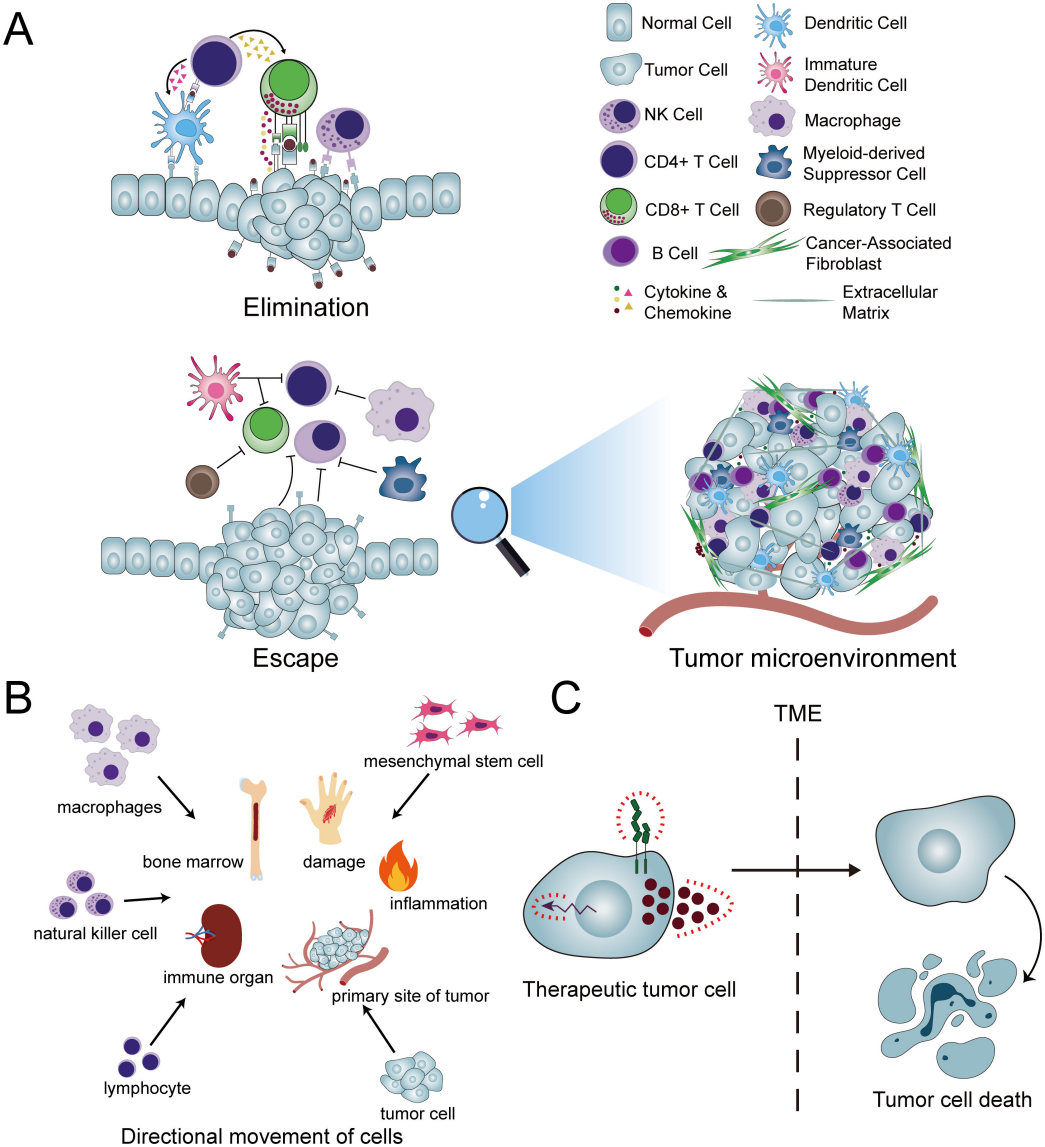
Concepts and applications pertaining to cellular homing. **(A)** In typical conditions, the innate and adaptive immune responses collaborate through a complex interplay of various immune cells and molecules to eliminate aberrant cells and tissues. However, within the tumor microenvironment (TME), this immune elimination is compromised. Consequently, the efficacy of therapeutic interventions such as CAR-T cells is significantly hindered, as these agents encounter difficulties in entering the tumor site and exerting their intended therapeutic impact. **(B)** A diverse array of cells exhibits homing capabilities, enabling them to migrate directionally to their respective target destinations. **(C)** The conversion of cancer cells into therapeutic tumor cells (ThTCs) and the exploitation of their inherent homing abilities circumvent the constraints imposed by the TME, thereby enabling the targeted elimination of cancer cells.

The homing of cancer cells is an intriguing aspect of cancer biology. Investigations have revealed that cancer cells, upon entering the circulatory system, can exhibit a propensity to migrate back to their original tumor site. For instance, cancer cells that detach from metastatic lesions not only recirculate to these secondary sites but may also reseed the primary tumor. This phenomenon, termed “cancer cell self-seeding,” has been well documented (9). Considering the potential implications, if cancer cells can be reprogrammed to function as anti-tumor agents, leveraging their inherent homing properties, we could potentially overcome the limitations imposed by the TME and deliver killer cells directly to the tumor site. Engineered cancer cells can target and eliminate tumors, including recurrent primary tumors and metastatic lesions in distant sites. This represents a promising therapeutic strategy for the treatment of cancer. At present, there are ongoing studies and explorations based on cell-homing principles. Noteworthy among them is attempting to exploit the homing behavior of cancer cells. However, at this stage, the research on this idea is not yet mature and has not yet reached scale. Utilizing tumor cells as “Trojan horses,” armed with therapeutic payloads, offers a novel approach to cancer therapy. This strategy, illustrated in **Figure 1**, is under development and holds significant promise for developing novel anticancer therapies and as a potentially transformative approach in oncology.

## 2 Cellular homing

**Figure 2:**
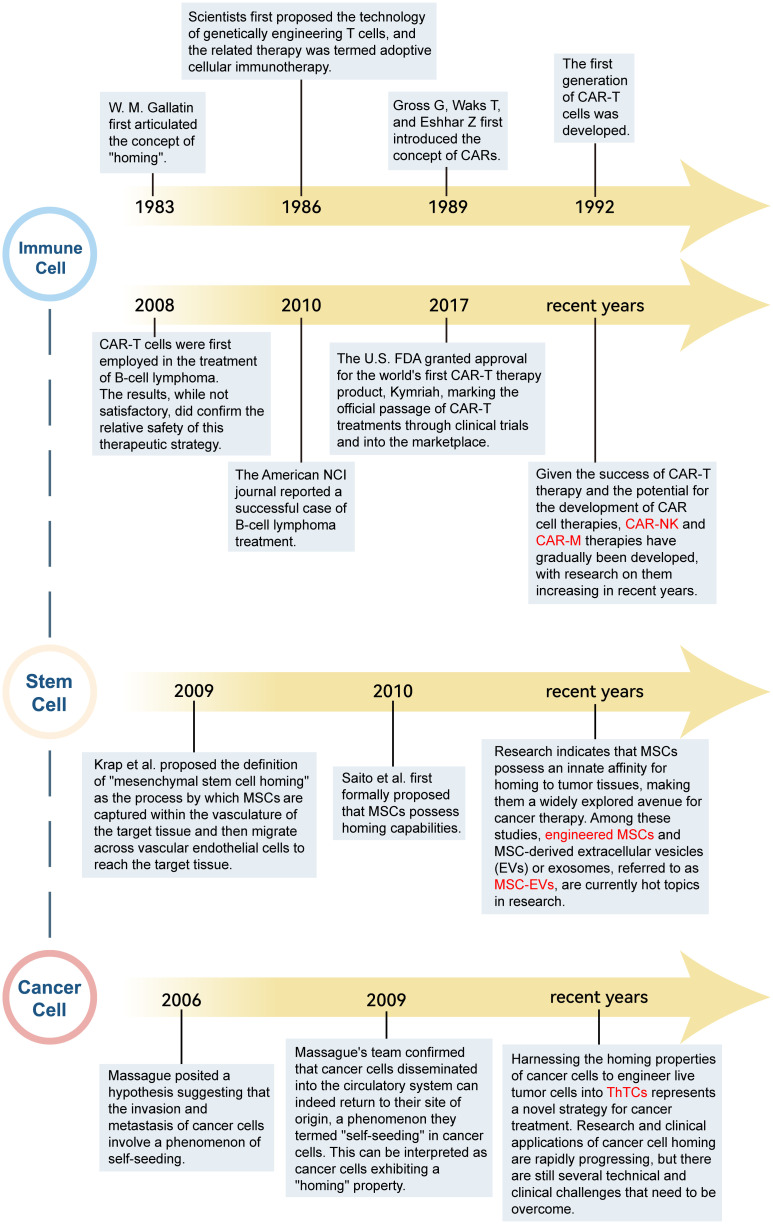
The development of cell-homing immunotherapy.

In the realm of biomedical research, an extensive array of investigations has been undertaken predicated on the tenets of cell homing theory, with a concerted effort to harness its principles for the advancement of immunotherapeutic strategies. The development of cell-homing immunotherapies is summarized in **Figure 2**. Among them, the two fields that have been studied more deeply include immune cells and stem cells.

### 2.1 Immune cell

Immune cell homing, a pivotal process within the immune system, is essential for maintaining its normal function and effectively responding to pathogens. Lymphocytes, a critical component of the immune system, are central to this process. In the context of an immune response, these lymphocytes migrate from lymphoid organs, such as the lymph nodes and spleen, to peripheral tissues, where they target and neutralize pathogens. Following the resolution of the immune response, lymphocytes must revert to specific immune organs, including lymphoid organs or the bone marrow—a process termed “homing.” This process involves various cell surface markers and subgroups, and the following are some specific immune cell subgroups and their associated surface markers that play a crucial role in in the homing effect (10–12). T Cells: T cells can be categorized into different subsets based on their surface markers and functions, such as CD4+ T cells and CD8+ T cells. These subsets may express different surface markers during the homing process, such as CD45RA and CD45RO, which are used to distinguish naive T cells from memory T cells. Naive T Cells: Naive T cells express lymph node homing receptors (CD62L) and chemokine receptors (such as CCR7), which are involved in the recirculation of naive T cells. Additionally, naive T cells highly express other cell surface markers, including CD27, CD28, and cell activation markers like CD44 and HLA-DR. Memory T Cells: Memory T cells are divided into at least two subsets based on homing characteristics and effector functions: Central Memory T Cells (TCM) and Effector Memory T Cells (TEM). TCM express CCR7 and CD62L, residing within lymphoid organs, while TEM possess distinct functions and migratory properties. Integrins: Integrins such as LFA-1, VLA-4, etc., play a significant role in T cell homing. For instance, VLA-4 (α4β1 integrin) is a key receptor for T cell homing to inflammatory sites, interacting with the vascular cell adhesion molecule VCAM-1. Selectins: Selectins such as L-selectin (CD62L) function in the homing of lymphocytes to peripheral lymph nodes. It binds to the peripheral node addressing (PNAd), mediating the adhesion of lymphocytes to the endothelial cells of peripheral lymph node vessels. Chemokine Receptors: Chemokine receptors such as CCR7 play a crucial role in T cell homing, especially during the homing process in lymph nodes. Other surface markers: Markers like CD44, CD27 are also involved in the homing process of immune cells. The specific combination of these subpopulations and surface markers enables immune cells to migrate specifically to the sites where they are required to exert their functions.

Besides lymphocytes, other immune cells, including natural killer (NK) cells and macrophages, play integral roles in the immune response and homing processes. NK cells, a vital subset of cytotoxic lymphocytes, exert a potent cytotoxic effect on tumor- and virus-infected cells. Analogous to lymphocytes, NK cells are also required to migrate from lymphoid organs to peripheral tissues and subsequently return to lymphoid organs following the completion of their functions (13). Macrophages, professional phagocytes responsible for the clearance of pathogens and cellular debris, adhere to a comparable migratory pathway.

Conclusively, the process of immune cell homing is a fundamental aspect of the immune system, facilitating the efficient localization and eradication of pathogens at infection sites, followed by the return of these cells to immune organs post-response. The intricate mechanisms and regulatory elements governing the homing of immune cells, including lymphocytes, NK cells, and macrophages, require in-depth investigation to fully elucidate their nuances.

### 2.2 Stem cell

The concept of stem cell homing encompasses the capacity of circulating or exogenous stem cells to identify and infiltrate specific environmental niches. This homing capability involves a variety of cell surface markers and subgroups, and below are some specific stem cell subgroups and their associated surface markers that play a crucial role in the homing effect. Mesenchymal Stem Cells: MSCs are multipotent stem cells that can differentiate into many cell types, including bone, fat, cartilage, muscle, and skin (14). The surface markers of MSCs include CD10, CD13, CD29, CD90, and CD105, while CD14, CD34, and CD45 are typically negative. These markers aid in the recognition and isolation of MSCs (15). Hematopoietic Stem Cells (HSCs): HSCs are multipotent cells capable of generating all types of blood cells. They produce red blood cells, white blood cells, platelets, and more through proliferation and differentiation. The surface markers of HSCs include CD34, CD38, CD90, CD117, CD123, etc. (16). Neural Stem Cells (NSCs): NSCs refer to those present in the nervous system, possessing the potential to differentiate into neuronal neurons, astrocytes, and oligodendrocytes. The surface markers of NSCs include Nestin, SOX2, ABCG2, FGFR1, and Frizzled-9, etc. (17). Cancer Stem Cells (CSCs): CSCs are a subset of cells characterized by self-renewal, differentiation potential, high tumorigenicity, and high drug resistance. The surface markers of CSCs include CD133 and CD44, etc. (18). Notably, the majority of research in this domain has been directed towards HSCs and MSCs (12, 19). HSCs are currently the best studied stem cells because stem cell transplantation relies on this process. This review, however, places a particular emphasis on the homing of MSCs, given their significance in tissue regeneration. MSCs reflect the innate ability of stem and progenitor cells to be recruited and become part of the tissue damage site that requires repair (20). As a subset of adult stem cells, MSCs are endowed with robust tissue differentiation and immune regulatory capacities, which are instrumental in the repair process. Their low immunogenicity, coupled with their propensity to home to diseased tissues, and their amenability to engineering, confer them with numerous advantages, making them a cell type of choice for therapeutic applications (21–24). Consequently, the utilization of MSCs in therapeutic contexts has been on the rise.

The cellular milieu encompassing stem cells constitutes a cradle-like environment, often termed the stem cell nest or niche. This niche serves not only as a source of sustenance for stem cells but also as a director of their behavior and a determinant of their differentiation trajectory (25, 26). Comprising a diverse array of cells in proximity to stem cells, the extracellular matrix (ECM), and an array of cytokines, the microenvironment plays a critical role in shaping the fate and function of stem cells (27, 28).

Stem cell homing involves a variety of molecular mechanisms (16), including interactions between vascular cell adhesion molecule-1 (VCAM-1) and integrin α4 (ITGA4), where these specific signaling molecules coordinate their migration behavior by interacting with corresponding receptors on the stem cell surface. Pioneer cells, such as VCAM-1-positive macrophages, play a guiding role in the homing process of stem cells, assisting in the localization of stem cells to specific microvascular structures. Variations within the microenvironment serve as primary catalysts for MSC homing, with distinct microenvironments secreting unique signaling molecules that selectively attract MSCs to targeted tissues. Extensive research has shown that the stem cell “niche,” a specialized microenvironment, modulates stem cell behavior through diverse signaling pathways. The surface of MSCs is replete with various receptors, including those for chemokines and growth factors, which facilitate MSC homing upon binding to their respective ligands. In ischemia, hypoxia, or tissue injury, the signaling molecules at the site of damage align with the MSC receptors, prompting the migration of endogenous or exogenously transplanted stem cells back into the microenvironment (2). Therefore, to enhance the efficacy of stem cell transplantation therapies, researchers must not only delve into the intrinsic properties of stem cells but also holistically assess the microenvironmental factors of the recipient tissue.

Broadly speaking, the process of stem cell homing constitutes a pivotal aspect of stem cell transplantation treatments. A thorough investigation into the mechanisms and regulatory factors governing stem cell homing is imperative for enhancing the therapeutic efficacy of stem cell interventions and fostering advancements in the realms of tissue regeneration and regenerative medicine.

### 2.3 Tumor cell

**Figure 3:**
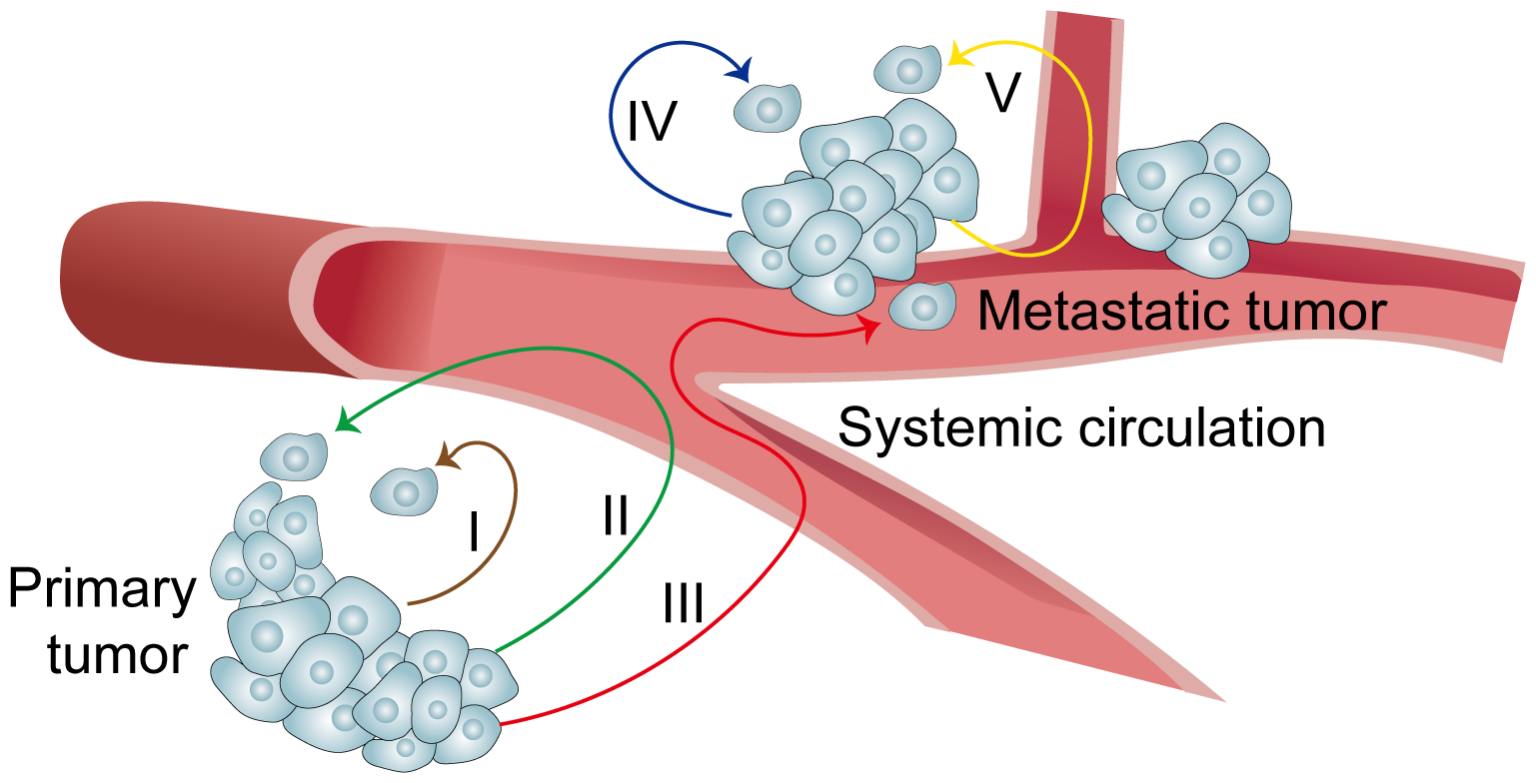
Summary of cancer cell migration pathways.

In addition to immune and stem cells, recent studies have revealed that cancer cells exhibit self-targeting capacity. The processes involved in cancer cell invasion and metastasis are exceedingly complex. Traditionally, cancer cell invasion has been found to follow pathways I (primary focus) and IV (metastasis), with metastasis occurring via route III (from primary to metastatic sites) (**Figure 3**). However, this unidirectional metastasis model (i.e., III) is inadequate to account for the various challenges in cancer treatment and the multifaceted biological characteristics of tumors (e.g., recurrence of primary tumors and limitations of targeted therapies). Consequently, researchers began to hypothesize the existence of unexplored pathways, such as II and V, during cancer cell invasion and metastasis, which involve the return of circulating cancer cells to their point of origin (29) (**Figure 3**). In 2009, Massague et al. confirmed that cancer cells disseminated into the circulatory system could reseed their original site. For instance, cancer cells released from metastases can return not only to metastatic sites but also to primary tumors. This phenomenon is termed “self-seeding” in cancer cells (9). In essence, cancer cells possess a form of “homing” ability. Research indicates that this homing is triggered by the TME. Moreover, cells that circulate and return to the primary site differ significantly from those that do not circulate. These self-seeding cells secrete substances that promote tumor growth and angiogenesis. Some investigators have further speculated that the dissemination of cancer cells into the circulation and their subsequent callback may be a mechanism by which tumors screen out stronger tumors (6).

## 3 Translational applications and regulatory factors of cell homing

With the advancements in medical technology, cell therapy has demonstrated considerable clinical potential for addressing various complex and challenging diseases, garnering significant interest among medical researchers. The initial focus on CAR-T cells for the treatment of malignant leukemia is not surprising, given the abundance of known antigens expressed on the cell membranes of blood cells and the relative ease of obtaining white blood cells and T cells that naturally target blood organs, such as blood, bone marrow, and lymph nodes (30). In 2017, the U.S. Food and Drug Administration (FDA) granted approval for the marketing of CAR-T therapies. Novartis’ Kymarih was the first to receive approval, indicated for the treatment of acute lymphoblastic leukemia (ALL) in pediatric and young adult patients. Clinical trials reported an objective response rate of 81% and a complete remission rate of 60% (31). Subsequently, Kite’s Yescarta became the second therapy to gain FDA approval, with indications for adult patients with recurrent or refractory large B-cell lymphoma, demonstrating an objective response rate of 82% and a complete response rate of 54%. Six months later, the complete remission rate remained at 36%, although one patient succumbed to a side effect of cerebral edema (32). To date, the FDA has approved five types of CAR-T cells, all targeting B-cell surface markers, with four targeting CD19 and one targeting the B-cell maturation antigen. All five therapies have been approved for the treatment of relapsed or refractory hematological malignancies, including B cell lymphoma, leukemia, and multiple myeloma (33).

CAR-T cells are highly valuable for tumor treatment, but significant challenges remain, especially with solid tumors (34). Solid tumor carcinoma cells originate from healthy tissues, predominantly healthy epithelial tissues, complicating the identification of a CAR-T target that is exclusively present in tumor cells. This challenge may result in CAR-T cell-induced toxic side effects in healthy tissues. Another impediment in the context of solid tumor CAR-T-cell therapy is the increased density of tumor tissues, which hinders T-cell infiltration. The third hurdle is the immunosuppressive microenvironment of solid tumors, which impedes CAR-T cell activation and proliferation.

In addition to T cells, the therapeutic potential of other immune cells, including NK cells and macrophages, has been investigated for cancer treatment. NK cell-based immunotherapies, particularly those using chimeric antigen receptor-modified NK (CAR-NK) cells, have demonstrated safety and efficacy (35). However, each CAR-modified immune cell therapy has unique advantages and limitations, and diverse CAR cell types can synergistically enhance tumor treatment outcomes (36). Apart from CAR cells, the concept of cell membrane camouflage has emerged as an innovative therapeutic approach. Certain studies have developed nanoparticles camouflaged with macrophage membranes, which harness the macrophage’s ability to precisely recognize antigens and target tumor sites (37).

Advancing the potential of cancer immunotherapy is a crucial frontier in oncological research. A limited understanding of the mechanisms of action of immune cells, immune resistance in the tumor microenvironment, and obstacles associated with delivering therapeutic agents *in vivo* must be addressed (38). The migration and homing of immune cells are governed by a complex interplay of various factors, including intracellular elements (e.g., genes and transcription factors), extracellular factors (including integrins, selectins, chemokines and their respective receptors, cytokine-induced signaling, and sphingosine-1-phosphate), and the cellular microenvironment (13, 39).

MSCs are adult pluripotent stem cells originating from a wide range of sources. Their utility in cancer therapy is enhanced by their inherent characteristics, including low immunogenicity, propensity to migrate to sites of tissue damage, paracrine effects, and immunomodulatory capabilities, coupled with their amenability to engineering manipulations. Despite ongoing controversies surrounding the application of MSCs in cancer treatment, evidence suggests that MSCs and their extracellular vesicles, which are engineered to overexpress anti-tumor genes (such as suicide genes, tumor necrosis factor, interleukins, and interferons) or are modified to deliver oncolytic viruses, nanoparticles, and anti-cancer drugs, can actively target tumor tissues and exert anti-tumor effects (40).

The migratory capacity of stem cells is a crucial determinant of the efficacy of stem cell transplantation. Nitzsche posited that MSC homing is a dynamic and ongoing process encompassing the mobilization of MSCs into the circulatory system, their migration across endothelial barriers, and their subsequent targeting of inflamed or damaged tissue regions (41). After introduction into the human body, exogenous MSCs can specifically target and exert restorative effects. Nonetheless, the clinical utility of MSCs is heavily contingent on their precise targeting of the injury site, with the number of homing cells significantly influencing their therapeutic potency. Enhancing the homing efficiency of MSCs remains a major challenge that must be addressed since their low targeting rates directly compromise clinical outcomes. Consequently, augmenting the homing efficacy of MSCs is imperative for therapeutic interventions. Current strategies to amplify MSCs’ homing capacity include genetic modifications, direct administration to target tissues, modulation of cell surface properties, *in vitro* activation of MSCs, reduction of intravascular retention in the lungs, implantation of chemokine- or cytokine-impregnated hydrogel scaffolds, application of pulsed ultrasound to injured tissues, combination of MSC membranes with a bioactive nanoparticle core, and magnetic guidance of MSCs treated with magnetic carbon nanotubes, among others (42–50). These methodologies have the potential to enhance the clinical efficacy of stem cell therapies to a significant degree.

## 4 Analysis of key elements in cellular homing therapy

Based on the current research progress, we endeavor to conduct an analysis of the relatively more mature clinical studies on the use of immune and stem cell homing therapies for cancer treatment. This analysis aims to help us identify and comprehend the needs and potential applications of cellular homing in cancer therapy, benefits and barriers that may promote or impede the adoption, and critical success factors that can guide the implementation of tumor treatment in clinical setting.

### 4.1 Needs and potential applications

In hematologic malignancies such as leukemia, myeloma, and non-Hodgkin B-cell lymphoma, adoptive CAR-T has demonstrated clinical efficacy in patients who are refractory to conventional chemotherapy. Nevertheless, the therapeutic utility of CAR-T in the context of solid tumors remains ambiguous. To address this, CAR-NK and CAR-macrophages (CAR-M) are emerging as potential alternatives or adjuncts to CAR-T cell therapy for solid tumor treatment. Concurrently, MSCs are a subject of intense research in the realm of cell-based therapies. The operability to engineer MSCs to secrete a variety of pro-apoptotic and anti-proliferative factors, coupled with their immunomodulation capacity and tumor-homing property, positions them as promising candidates for the treatment of diverse malignancies (51). In specific cases of brain cancers, such as glioblastoma multiforme (GBM), MSC transplantation offers a strategy for the localized delivery of therapeutic molecules that are otherwise impermeable to the blood-brain barrier when administered systemically (52–54).

### 4.2 Benefits and barriers

The use of CAR T-cell therapy in the management of solid tumors is predominantly hindered by various challenges, including restricted tumor trafficking and infiltration, the existence of an immunosuppressive tumor microenvironment, and the occurrence of adverse events associated with this modality. CAR-NK cells are promising alternatives to CAR-T cells owing to their independence from human leukocyte antigen (HLA) compatibility and reduced toxicity. Moreover, CAR NK cells can be generated in large quantities from various sources, rendering them potential off-the-shelf therapeutic agents. Despite the evident benefits of CAR NK cell therapy over CAR T cell therapy, significant constraints persist. Most of the limitations associated with CAR-T therapy also apply to CAR-NK cells, including challenges related to NK cell migration to tumor sites and adverse tumor microenvironments. Additionally, the short half-life of NK cells (<10 days) (55) presents a dual-edged dynamic in the context of CAR-NK therapy, offering a safety advantage in scenarios of severe toxicity, yet necessitating repeated dosing to elicit a sustained response. CAR-M therapy addresses several pivotal hurdles faced by contemporary CAR-T cell therapy by merging the innate and adaptive immune responses to orchestrate a multifaceted offense against tumors. Nevertheless, CAR-M therapy remains in the preliminary stages, with only one clinical trial (Clinicaltrials.gov identifier number: NCT04660929), the results of which remain to be reported. Consequently, numerous limitations may yet to be uncovered. Similar to CAR-T and NK cells, CAR-M must navigate through the seven stages of the cancer immune cycle to elicit cytotoxic effects.

MSCs inherently possess the capacity to migrate toward a diverse array of chemokines secreted by tumor tissues or their microenvironment, facilitating their targeted homing to neoplastic foci. This unique attribute makes MSCs innovative living vectors for the delivery of anti-tumor agents and genetic materials. MSC-mediated delivery systems are capable of the targeted transport of chemotherapeutic agents, including doxorubicin, paclitaxel, and gemcitabine, thereby overcoming issues of the short half-life of these drugs and their inadequate tumor-specific targeting (56, 57). Additionally, MSC can effectively protect and target the delivery of therapeutic genes, such as tumor cell-killing genes and immune system regulatory genes, through genetic recombination and achieve tumor suppression or killing effects by specifically expressing therapeutic genes at the tumor site (58). Nonetheless, despite the evident benefits of MSC-based therapies, several inherent and extrinsic challenges curtail their broader clinical application: (1) The *in vitro* growth rate of adult MSCs is generally low unless artificially immortalized; (2) the limited replicative potential, which complicates the engineering of therapeutic molecules and diminishes efficacy due to reduced viability *in vivo* (59); (3) the potential mismatch of donor MSCs, derived from healthy individuals or donor banks, with the recipient’s HLA status, which may induce adverse immune responses and/or toxicity, as well as premature immune clearance of MSCs by the recipient (60); (4) while autologous MSC transplantation represents an optimal approach, it is time-intensive and currently impractical for first-line therapy or patients with advanced-stage cancer, as it requires the harvesting, reengineering, and expansion of MSCs prior to reinfusion (61). Furthermore, harvesting MSCs from patients necessitates additional invasive procedures, thereby enhancing the overall risk of clinical complications, particularly in advanced-stage and immunocompromised patients after chemotherapy.

### 4.3 Critical success factors

We identified the pivotal determinants of successful homing therapy, which predominantly encompass the following facets: (1) The efficiency of cell migration and infiltration into tumors, which represents the paramount consideration. Killer cells must effectively localize at the tumor site to target and eliminate cancerous cells within the lesion. (2) Countering the tumor microenvironment: It is imperative that, once within the tumor site, therapeutic cells maintain their functionality and are uninhibited by the TME. (3) Antigen evasion and downregulation: Despite initial therapeutic efficacy, the challenges of cancer cell antigen escape and downregulation must be addressed. (4) Killer cell availability, expansion, and persistence: Optimal therapeutic candidates should be readily accessible and capable of stable expansion, and their persistence in the body significantly influences treatment approaches. (5) Safety: Therapeutic intervention must not only be efficacious but also ensure sufficient safety and be devoid of systemic toxicity and adverse effects. (6) Resistance to immune clearance and controllability: Therapeutic cells must resist elimination by the immune system before exerting their therapeutic impact. However, these effects must be controlled and should not cause secondary cancers.

## 5 Utilizing the self-homing of cancer cells to treat tumors: a novel approach

Immunotherapy is currently at the forefront of cancer treatment; however, the population that truly benefits from immunotherapy remains limited. One of the main reasons for this is that the tumor microenvironment is very powerful, suppressed immune cells are not easily activated, and it is not easy for killer CAR-T cells and engineered MSCs to enter tumors. Researchers have explored numerous strategies to enhance the homing efficacy of immune cells and stem cells at tumor sites. A novel approach that has garnered attention in recent years involves leveraging the inherent homing properties of cancer cells and modifying them to improve the targeting of therapeutic cells to tumor tissues.

Three principal challenges are encountered when selecting cancer cells as “killer” candidates. First, what mechanisms can be employed to program these modified cells to “prioritize justice over familial allegiance”? Second, despite these modifications, the cells remain cancerous. How to ensure that one’s actions are benign? Third, how can safety be guaranteed before deployment for tumor eradication? Addressing these three questions simplifies the therapeutic process.

Using inactivated tumor cells can elicit potent anti-tumor immune responses. However, the effectiveness of this approach is constrained by its inability to eliminate tumor cells prior to the initiation of an immune response and its limited or no clinical benefit (62–64), which may be attributed to the lack of direct cytotoxic effects on tumor cells and the inability to elicit strong anti-tumor immune responses. Unlike inactivated tumor cells, viable tumor cells have a unique potential for self-homing/-targeting (29). Therefore, engineering tumor cells to express therapeutic agents is a logical approach to exploit neoantigens from their natural origin. If the cells in the body can be summoned back by themselves, then only cancer cells can be obtained from the patient’s body (such as obtained during surgery), modified and injected into the circulatory system. This will not only attack the primary lesions, but also eliminate the metastases.

**Figure 4:**
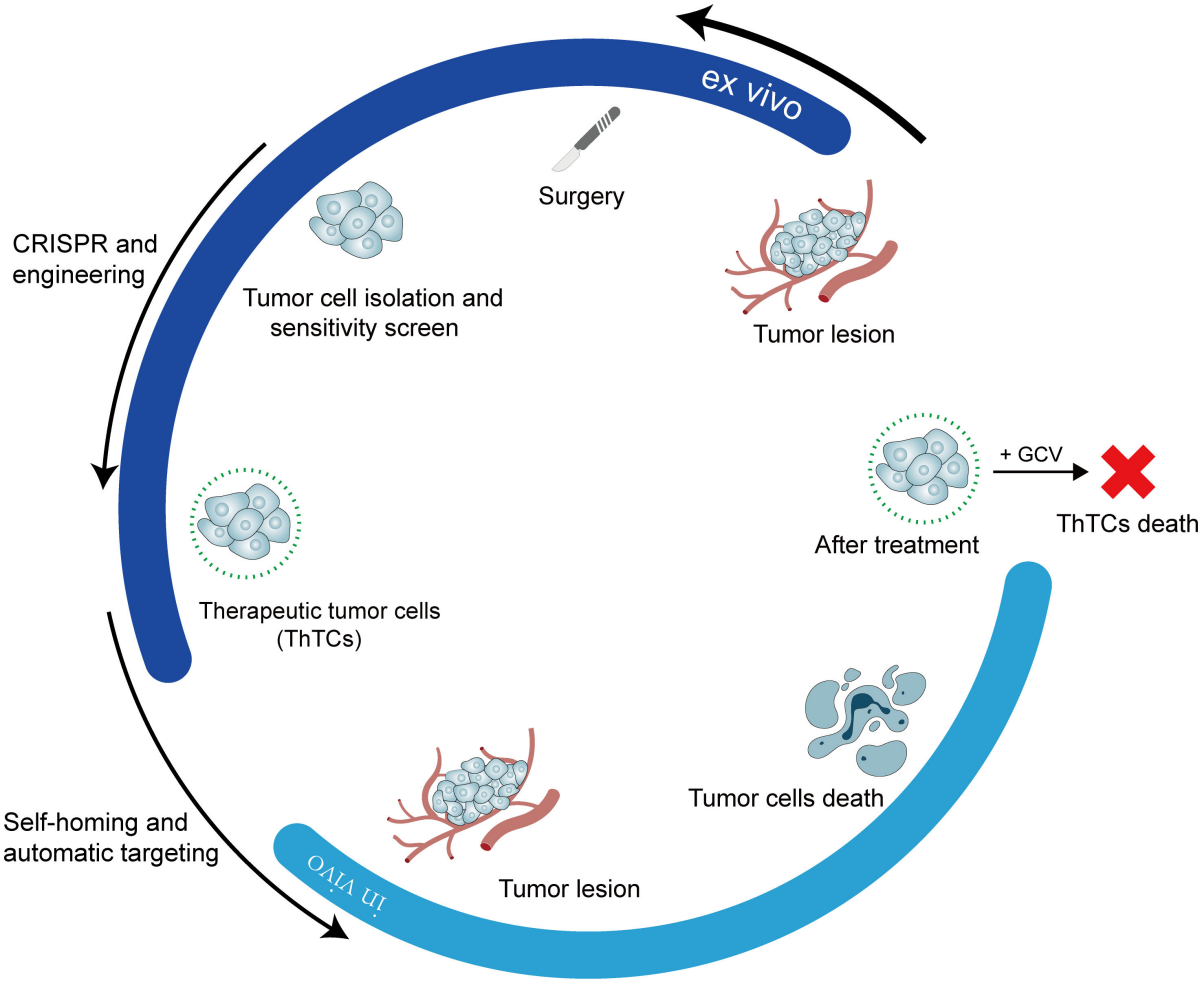
Summary of cancer treatment strategies based on tumor cell homing.

Dr. Shah’s research team put this concept into practice and identified a ligand molecule, such as interferon-beta (IFN-β), capable of binding to specific receptors on the surface of various cancer cell types, thereby triggering apoptosis. Subsequently, within a laboratory environment, a cancer cell lacking the aforementioned death receptor was identified, thereby safeguarding the cell from autolysis by its ligand. The gene encoding the death-inducing ligand molecule was transduced into the cells. Consequently, cancer cells possess a mechanism to “eliminate kin” while ensuring survival. The subsequent problem to be resolved involves engineering these cells to induce their death following the targeted elimination of their relatives. Given this, investigators have engineered cancer-killing cells by integrating a gene encoding an enzyme capable of synthesizing a prodrug that converts ganciclovir (GCV) into a cytotoxic agent effective against cancer cells (**Figure 4**). At this point, the three key issues have been resolved. The efficacy of this strategy was evaluated in mouse models of highly malignant glioblastoma and breast cancer. The post-injection of genetically modified killer cancer cells into mouse tumors targeted both primary and metastatic lesions, resulting in tumor regression. After treatment with GCV, mouse survival time was significantly extended (65). Therapeutic efficacy *in vivo* is contingent upon not only the direct cytotoxic action on tumors but also the anti-tumor immune response triggered by therapeutic tumor cells (ThTCs). Moreover, cancer cells resistant to death ligands may be eliminated through a bystander effect mediated by the surrounding killer cells (66).

The potential benefits of the aforementioned cancer cell-based therapy, when compared to CAR cell- and MSC-based approaches, include the enhanced targeting of therapeutic cancer cells to the tumor site, facilitated engineering with therapeutic molecules owing to their robust *in vitro* growth, and prolonged survival of therapeutic cells, which collectively contribute to enhanced *in vivo* therapeutic efficacy. Additionally, the ready availability of these cells for autologous therapies, given their procurement from tumor biopsies, a routine examination for most cancer patients, further supports their clinical utility. Treatment with autologous cells offers an advantage over allogeneic approaches by mitigating the risk of adverse immune responses and/or toxicity, as well as the potential for premature clearance of therapeutic cells by the recipient’s immune system. Adapting ThTC strategies to primary solid tumors remains challenging, given the time required to engineer autologous cell lines. Therefore, in the current context, it is appropriate to treat recurrent or metastatic disease with ThTCs.

Overall, Dr. Shah’s research introduced novel perspectives on cellular cancer therapies. Recent endeavors have explored the utilization of the inherent homing properties of tumor cells to target the delivery of killers to the primary tumor site. Prominent among these strategies are the use of tumor cells as vectors for oncolytic virus delivery (67, 68), the application of engineered tumor cells expressing suicide genes to convey death signals to neighboring tumor cells (the bystander effect) (69, 70), and targeting TME through the engineering of cancer cells to express therapeutic agents that influence the tumor neovascular endothelium (71). However, the proposed methods are marginally bold. First, they involve modifying cancer cells, culminating in injecting live cancer cells into treated individuals. Although safety mechanisms exist, the potential for secondary malignancies warrants careful consideration. Second, the path to clinical applications, including the integration of related CRISPR gene editing technologies, is fraught with challenges. Indeed, < 5% of experimental cancer therapies are authorized by the FDA (72). However, this does not deter efforts in cancer cell-homing therapy, as numerous promising research outcomes have been attained within this domain.

## 6 Conclusion and perspectives

Recently, there has been tremendous progress in the clinical development of cell-homing therapies for cancer treatment, especially for solid tumors. However, some challenges faced by mainstream CAR-T cell therapy are related to the tumor microenvironment, such as the lack of tumor-specific antigens, inefficiency of CAR-T cell trafficking and migration to the tumor site, and the presence of an immunosuppressive TME. Concurrently, CAR-NK cells and engineered MSCs have been the subject of extensive investigation and have entered clinical trials. CAR-NK cells exhibit several advantages, including a reduced risk of on-target/off-tumor toxicity due to their limited lifespan, diminished likelihood of cytokine release syndrome and neurotoxicity due to distinct cytokine profiles, and the ability to be derived from various sources, thereby mitigating the risk of alloreactivity. Additionally, CAR-NK cells can engage tumors in both CAR-dependent and -independent manners, a unique attribute of NK cells. Nevertheless, challenges similar to those associated with CAR T cells, such as infiltration into the tumor tissue and resistance to the immunosuppressive microenvironment, have also been observed in CAR-NK cells. Utilizing the low immunogenicity, homing properties, and paracrine and immunomodulatory abilities of MSCs, they can be transformed into efficient transport vehicles for carrying anti-tumor information molecules to target tumor cells or the microenvironment. Moreover, MSCs have the potential to prevent or alleviate graft-versus-host disease. In summary, the engineered MSCs exhibited superior anti-tumor effects. However, unmodified MSCs exhibit dual effects, potentially promoting or suppressing tumor growth. Some studies have indicated that MSCs may promote tumor development through immunosuppression, secretion of factors that stimulate tumor growth and invasion, or enhancement of tumor angiogenesis (73–76). Conversely, other researchers suggest that MSCs can inhibit the development of melanoma, pancreatic cancer, pancreatic ductal adenocarcinoma, and bone metastasis of prostate cancer (77–80). In view of this, the anti-tumor safety and effectiveness of natural MSCs cannot be controlled precisely.

CAR-M and ThTC are regarded as more potential anti-tumor therapeutic modalities. Macrophages are one of the primary infiltrating cells of the TME, and CAR-M cells can function through both the innate and adaptive immune systems. Preclinical investigations have demonstrated encouraging anti-tumor efficacy. Future CAR M therapies still need to overcome some of the obstacles encountered by CAR-T therapies. Given the self-targeting capabilities of living tumor cells, which can autonomously localize and migrate to both primary and metastatic sites, designing tumor cells as ThTCs is a novel and innovative approach. To date, numerous compelling research outcomes have been achieved in animal models, with ongoing efforts to translate these findings into clinical applications.

Tumor cells are astutely adaptive, exploiting the human body’s resources to an extreme extent. They facilitate the provision of energy and detoxification by tumor-associated fibroblasts, enabling cancer cells to endure the challenging environment within the tumor (81). In addition, tumor cells co-opt macrophages to assist in early-stage metastasis (82). Furthermore, during metastasis, some cancer cells harbor microorganisms, such as Fusobacterium, which aid in establishing cancer cells in various body locations (83). Therefore, cancer treatment remains a significant challenge.

Prior to the extensive incorporation of immunotherapy, outcomes for many advanced cancer cases are a few more months of life. Immunotherapy holds the potential for achieving complete tumor eradication, marking a significant advancement in cancer treatment. Although the response rates are not exceptionally high, for those patients who respond, the therapeutic effect is transformative.

Overall, therapeutic approaches that leverage the homing properties of cells exhibit considerable potential; however, current clinical outcomes are yet to meet expectations. Researchers are actively exploring strategies to enhance the efficacy of cell-homing therapies, which include integrating advanced technologies, such as artificial intelligence and nanotechnology. These innovations are poised to surmount several limitations inherent in conventional cell-based therapies. Furthermore, the judicious integration of cell-homing therapies with conventional cancer treatment modalities is anticipated to bolster anti-tumor efficacy. For instance, after the resection of the primary tumor, actively proliferating cancer cells derived from the patient’s own tissue can be engineered into ThTCs for the management of recurring or metastatic disease. Looking ahead, a heightened focus on the engineering of ThTCs is warranted, including the exploration of their underlying CRISPR gene-editing technologies, to optimize therapeutic efficacy, mitigate the risk of secondary tumorigenesis, and facilitate their application in treating primary solid tumors. Significantly, in-depth clinical research remains necessary to support engineered ThTC-targeted therapy for tumors to broaden the clinical application prospects of ThTC.
